# Expression of c-Fos and c-Jun in adjacent cervical spinal cord segments following C_7_ nerve root rhizotomy in rats: Indication of a neural pathway between adjacent cervical spinal cord segments

**DOI:** 10.3892/etm.2013.1136

**Published:** 2013-06-03

**Authors:** HUI LI, QING LI, KELIANG XIE, SHIQING FENG, PEI WANG, XINLONG MA

**Affiliations:** 1 Department of Orthopedic Surgery, Tianjin Medical University General Hospital, Tianjin 300052, P.R. China; 2 Tianjin Institute of Anesthesiology, Department of Anesthesiology, Tianjin Medical University General Hospital, Tianjin 300052, P.R. China

**Keywords:** cervical radiculopathy, neural pathway, c-Fos, c-Jun, spinal cord

## Abstract

Cervical radiculopathy is a common disease in clinical practice. However, the symptoms are not confined to the affected spinal cord segment indicated by magnetic resonance imaging (MRI) findings. In the present study, we measured c-Fos and c-Jun expression in ipsilateral and adjacent cervical spinal cord segments following C_7_ nerve root rhizotomy, to determine whether there is a neural pathway between adjacent cervical spinal cord segments. Forty-eight adult male Wistar rats were randomly divided into two groups: the C_7_ rhizotomy group (rhizotomy group, n=24) and the sham-operated group (sham group, n=24). The right C_7_ nerve root was completely cut off in the rhizotomy group, while it was exposed but not cut in the sham group. The expression of c-Fos and c-Jun in cervical spinal cord segments was detected by immunohistochemistry at 2 and 4 h after surgery. We observed that the number of c-Fos- and c-Jun-positive neurons in ipsilateral C_5–7_ segments were significantly increased at 2 and 4 h after C_7_ nerve root rhizotomy (P<0.05 vs. the sham group). The location of c-Fosand c-Jun-positive neurons in C_5–7_ gray matter was similar in the rhizotomy and sham groups, which was mainly in lamina IX of the anterior horn and laminae I–II of the dorsal horn of the spinal cord. However, the number of c-Fos- and c-Jun-positive neurons in the C_5–7_ gray matter was significantly reduced at 4 h after surgery compared with the number 2 h after surgery. The location of c-Fos- and c-Jun-positive neurons at 4 h was similar with that at 2 h. Therefore, there may be a neural pathway between ipsilateral adjacent cervical spinal cord segments. This may be one possible explanation as to why the radicular symptoms of cervical radiculopathy are not confined to the affected spinal cord segment shown by MRI.

## Introduction

Cervical radiculopathy is a common disease manifesting as radiated pain, weakness or numbness of the upper extremities and stiffness or limited motion of the neck (1). However, the syndrome of cervical radiculopathy often affects areas far beyond the innervation area of the affected nerves and is not consistent with the magnetic resonance imaging (MRI) findings, which makes identification of the location of the disease and treatment difficult and may even lead to mismanagement (1). The explanation of this phenomenon remains unclear.

c-Fos, the protein product of immediate-early gene (IEG) *c-fos*, has been widely used as a tool for the study of neural correlates of nociception (2–5) and as a marker for neuronal activation following noxious stimulation. c-Jun, the protein product of another IEG *c-jun*, is also reported to be a marker for neuronal activation and noxious stimulation (6,7).

We hypothesize that there are neural pathways between adjacent segments of the cervical spinal cord. This hypothesis may explain the mismatch between the symptoms and the affected spinal cord segment. In order to test our hypothesis, the present study was designed to investigate the expression of c-Fos and c-Jun in ipsilateral C_5–7_ segments following unilateral C_7_ nerve root rhizotomy in rats. The findings may offer a possible explanation for this clinical phenomenon.

## Materials and methods

### Animals

All experiments were performed according to the Guidelines on Ethical Standards for Investigation of Experimental Pain in Animals (Zimmermann, 1983). Experiments were performed on adult male Wistar rats (220–250 g). All animals were provided by the Experimental Animal Center of Tianjin Medical University (Tianjin, China) and housed in groups of 2 or 3 in clear plastic cages. Food and water were freely available during this study. Animals were randomly divided into two groups: i) the C_7_ rhizotomy group (rhizotomy group, n=24), for which animals received right C_7_ nerve root rhizotomy and ii) the sham-operated group (sham group, n=24), for which animals underwent the same surgery without right C_7_ nerve root rhizotomy. Each group was subdivided into two subgroups (2 and 4 h after surgery).

### Surgery

All the animals were intraperitoneally anesthetized with chloral hydrate (300 mg/kg). An incision was made in the middle of the back and the skin and superficial muscle were retracted. A laminectomy was performed from C_6_ to T_1_. During surgery, the surgeon was extremely careful to avoid any damage to the cervical spinal cord. The right C_7_ nerve root was exposed by the operating microscope and then broken completely. The incision was carefully closed.

### c-Fos and c-Jun immunohistochemistry

At 2 and 4 h after surgery, the animals were deeply anesthetized with an overdose of chloral hydrate and underwent transcardial perfusion with 150 ml normal saline (4°C), followed by 4% paraformaldehyde in 0.1 M phosphate-buffered saline (PBS; pH 7.4). The C_5–7_ segments of the spinal cord were removed and post-fixed in 4% paraformaldehyde containing sucrose (10%) at 4°C for 1 h and then transferred to PBS containing sucrose (30%) overnight. Then, the spinal cord segments were serially sectioned at 10 *μ*m thickness in a transverse plane with a freezing microtome, and one from every eight serial sections was collected and processed for c-Fos and c-Jun immunohistochemistry.

For c-Fos or c-Jun immunostaining, a standard avidin-biotin-peroxidase complex (ABC) technique was performed using a Histostain SP kit (Boshide Biological Technology Co., Wuhan, China) according to the manufacturer’s instructions. Briefly, free-floating sections were incubated overnight in a rabbit monoclonal anti-c-Fos or c-Jun antibody diluted in PBS (1:300). After several washes in PBS, the sections were incubated in biotinylated goat anti-rabbit IgG diluted to 1:200 in PBS for 20 min, rinsed in PBS and incubated with avidin-biotin reagents (1:100) for 20 min at room temperature. After three washes in Tris-buffered saline (TBS), sections were developed in 3,3′-diaminobenzidine tetrahydrochloride solution containing 0.05% H_2_O_2_ in TBS for 30 min. Sections were then washed in distilled water, mounted on slides, air-dried, dehydrated through graded ethanol solutions followed by xylene and then coverslipped with Permount for cell counting under a light microscope.

### Image analysis and quantification

**Figure 1.**
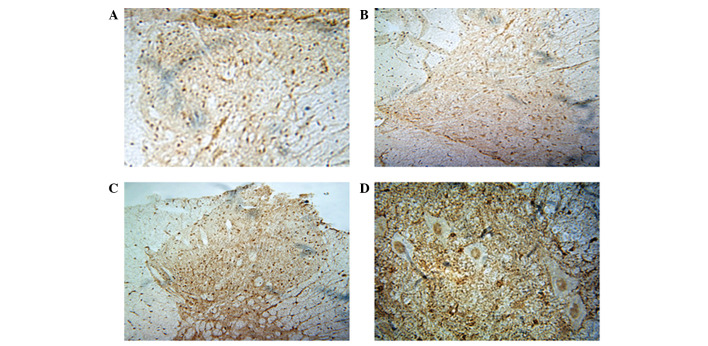
c-Fos immunoreactive neurons in right cervical spinal cord segments following rhizotomy. c-Fos-positive neurons observed following right C_7_ rhizotomy in spinal grey matter of right (A) C_5_, (B) C_6_ and (C) C_7_ segments (magnification, ×100). (D) c-Fos-positive neurons in spinal grey matter (magnification, ×400). The avidin-biotin complex (ABC) method was used.

Analysis of c-Fos or c-Jun immunoreactivity was quantified by determining the number of c-Fos- or c-Jun-positive neurons in the right spinal gray matter. The regions of the gray matter corresponded to Rexed’s superficial laminae I–III and deeper laminae IV–VI. The neurons were considered as c-Fos- or c-Jun-positive if the immunostaining of their nuclei was brown and were considered negative if the staining within the nuclei was at background levels (Figs. 1 and ). Tissue sections were examined using a CAS Immunohistochemistry Image Analysis System (CAS, East Rutherford, NJ, USA). Four sections in one spinal cord segment were randomly selected for counting of c-Fos- or c-Jun-positive neurons, respectively, in a high magnification field. The c-Fos- or c-Jun-positive neurons were recorded in three groups at the different time-points from C_5–7_ right spinal gray matter, respectively.

### Statistical analysis

All data are expressed as mean ± SEM. Statistical analysis was performed using SPSS 13.0 (SPSS, Inc., Chicago, IL, USA) for Windows. Statistical significance was calculated by the t-test. P<0.05 was considered to indicate a statistically significant difference.

## Results

In the present study, we identified that the expression of c-Fos and c-Jun in control animals was extremely weak, with only small numbers of c-Fos- and c-Jun-positive neurons scattered in the grey matter (data not shown).

**Figure 2.**
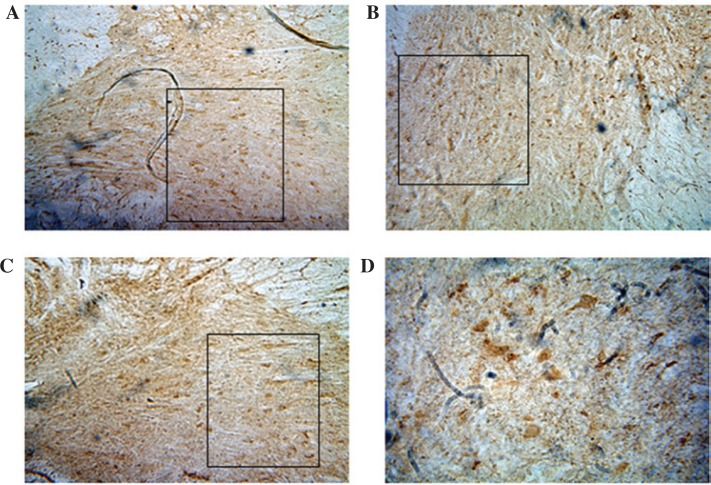
c-Jun immunoreactive neurons in right cervical spinal cord segments following rhizotomy. c-Jun-positive neurons observed following right C_7_ rhizotomy in spinal grey matter of right (A) C_5_, (B) C_6_ and (C) C_7_ segments (magnification, ×100). (D) c-Jun-positive neurons in spinal grey matter (magnification, ×400). Black boxes show the c-Jun-positive neurons. The avidin-biotin complex (ABC) method was used.

**Figure 3.**
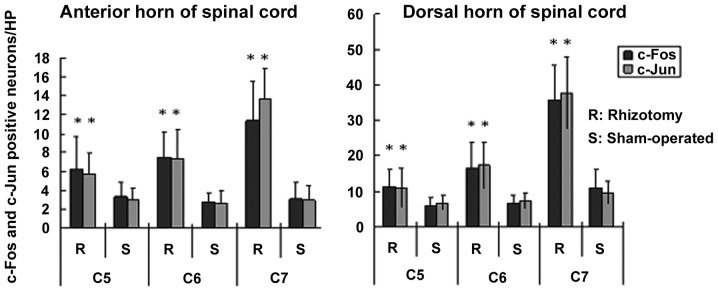
Number of c-Fos- and c-Jun-positive neurons at 2 h after surgery. The numbers of c-Fos- and c-Jun-positive neurons increased significantly in the anterior horn and dorsal horn of the spinal cord in all three segments following rhizotomy compared with those in the sham-operated group. There was no difference between the number of c-Fos- and c-Jun-positive neurons at each location in each group. ^*^P<0.05 vs. the sham-operated group. HP, high power field.

**Figure 4.**
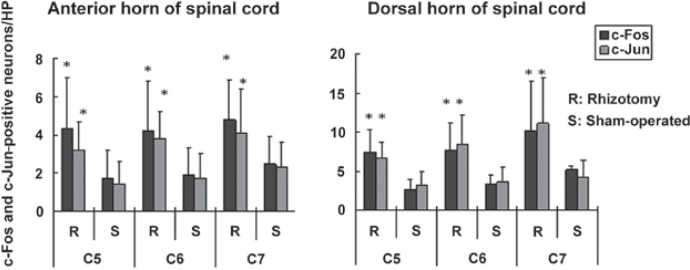
Number of c-Fos- and c-Jun-positive neurons at 4 h after surgery. The numbers of c-Fos- and c-Jun-positive neurons increased significantly in the anterior horn and dorsal horn of the spinal cord in all three segments compared with those in the sham-operated group. There was no difference between the number of c-Fos- and c-Jun-positive neurons at each location in each group. ^*^P<0.05 vs. the sham-operated group. HP, high power field.

**Figure 5.**
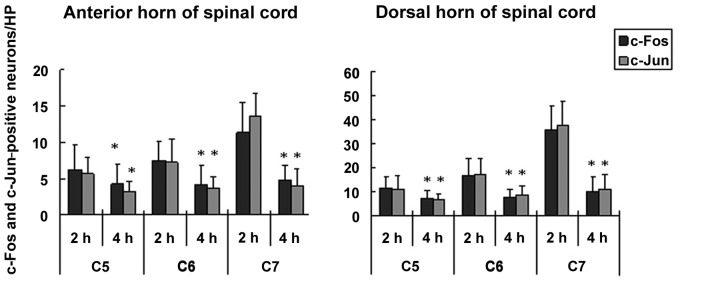
Number of c-Fos- and c-Jun-positive neurons at different time-points after rhizotomy. The numbers of c-Fos- and c-Jun-positive neurons markedly decreased at 4 h after surgery in the anterior horn and dorsal horn of the spinal cord in all three segments compared with those 2 h after surgery, t. ^*^P<0.05 vs. 2 h after rhizotomy. HP, high power field.

In the rhizotomy and sham-operated groups, c-Fos- and c-Jun-positive neurons were visible not only in right C_7_, but also in ipsilateral C_5–6_ segments of the spinal cord at 2 and 4 h after surgery (Figs. 1 and 2). The numbers of c-Fos- and c-Jun-positive neurons in the rhizotomy group were markedly increased compared with those in the sham group, at the same time-point and in the same segment (P<0.05; Figs. 3 and 4). The location of these neurons was similar in the rhizotomy and sham groups, which was mainly in Rexed’s lamina IX (anterior horn of the grey matter) and in Rexed’s laminae I–II (dorsal horn of the grey matter) of the cervical cord. Moreover, in the rhizotomy group, the numbers of c-Fos- and c-Jun-positive neurons in the anterior horn and posterior horn of the grey matter were significantly lower at 4 h after surgery than at the 2 h time-point (P<0.05; Fig. 5). The location of c-Fos- and c-Jun-positive neurons was similar at 2 and 4 h after surgery.

## Discussion

Cervical radiculopathy is a clinical manifestation of degenerative cervical spine disease and commonly occurs in clinical practice (1). The diagnosis is usually based on the symptoms, signs and imaging, particularly using MRI, which offers strong evidence for diagnosis following careful history taking and physical examination (8). However, occasionally the motor (weakness and atrophy), sensory (pain or paresthesias) and reflex (diminution or absence of tendon reflexes) symptoms are not confined to the affected spinal cord segment that corresponds with the MRI findings (1). Therefore, the precise location of the affected spinal cord segment becomes confused, leading to difficulties in creating a treatment plan. In the present study, we aimed to determine whether there is a neural pathway between adjacent segments of the cervical spinal cord in rats and to provide a possible explanation for the mismatch of the symptoms and imaging results.

The IEG *c-fos*, which is rapidly and transiently expressed in neurons in response to stimulation, transcribes the nuclear protein c-Fos in spinal cord neurons following induction of gene transcription (5,9). c-Fos immunoreactivity has been widely used as a functional marker to identify activity in spinal neurons in response to noxious stimulation (3–5) and provides a good technique for efficiently visualizing the individual cells activated by or associated with noxious input (2). A number of studies have shown that various types of noxious stimulation, including thermal, mechanical and chemical stimuli (10,11), and neuropathic pain models, including constriction injury (2,12), as well as spinal cord stimulation (13), induce the expression of c-Fos in the spinal cord. Sugimoto *et al* also reported that c-Fos expression is induced in the rat spinal dorsal horn following L_5_ dorsal root rhizotomy (14). c-Jun, the protein product of *c-jun*, another IEG, is also reported to be a marker for neuronal activation and noxious stimulation (6,7). Furthermore, Ke *et al* demonstrated that partial dorsal root rhizotomy led to the upregulation of *c-jun* expression in neurons of the dorsal root ganglion (15). The duration of *c-fos* and *c-jun* expression varied from several minutes to several days in different models (9,16), which was a considerable discrepancy. In the current study, the c-Fos- and c-Jun-positive neurons were detectable in the ipsilateral C_7_ segment at 2 h after C_7_ nerve root rhizotomy.

In the present study, in addition to the C_7_ segment, an evident amount of c-Fos- and c-Jun-positive neurons was also observed in C_5_ and C_6_ segments of spinal grey matter following right C_7_ nerve root rhizotomy, and the number of positive neurons was significantly increased compared with that in the sham-operated animals in the same segment and same time-point. These positive neurons had a similar location, which was in Rexed’s lamina IX in the anterior horn and in Rexed’s laminae I–II in the dorsal horn of the spinal cord. Although the early increase of c-Fos immunoreactivity may contain a component of *c-fos* expression through surgical injury and inflammation (17), the surgical procedures were identical, with the exception that the C_7_ nerve root was exposed in the sham-operated group and was severed in the rhizotomy group. Therefore, the difference in c-Fos expression in the same segment between the two groups was mainly caused by the right C_7_ rhizotomy. The same was observed with c-Jun expression. This study demonstrates that C_7_ nerve root rhizotomy triggers c-Fos and c-Jun expression in ipsilateral C_5_ and C_6_ segments of the cervical cord. These findings indicate that the afferent sensory fibers of the C_7_ nerve root project not only to the dorsal horn of the ipsilateral C_7_ spinal cord, but also to at least two adjacent segments (C_6_ and C_5_ segments). In addition, the efferent motor fibers of C_7_ nerve roots are composed of fibers coming from not only the ipsilateral C_7_ segment, but also from at least two adjacent segments (C_6_ and C_5_). Therefore, there must be a neural pathway between adjacent segments of the cervical spinal cord.

Furthermore, 4 h after right C_7_ rhizotomy, the c-Fos- and c-Jun-positive neurons were still visible in C_5–7_ segments. The number of c-Fos- and c-Jun-positive neurons in the rhizotomy group was significantly increased in the anterior horn and dorsal horn of the spinal cord compared with the number in the sham-operated group,. However, compared with that at 2 h after rhizotomy, the number of c-Fos- and c-Jun-positive neurons had decreased considerably in all three segments. However, the location of these positive neurons did not change between the 2 and 4 h time-points. Following C_7_ rhizotomy, expression of IEGs was observed and then the expression reduced simultaneously in all C_5–7_ segments. The expression of IEGs in C_5_ and C_6_ presented the same trend as that in C_7_, which suggests that there were neurons from C_5_ and C_6_ segments that were activated in response to ipsilateral C_7_ rhizotomy in the same way as the C_7_ neurons were. This indicates that a number of neurons in C_5_ and C_6_ also contribute to the construction of the C_7_ nerve root. This provides a possible explanation as to why the radicular symptoms are not confined to the affected spinal cord segment as shown by MRI.

In order to provide stronger evidence, we selected two IEGs, *c-fos* and *c-jun*. These are important research tools in the study of the neural basis of stimuli and injury (9). In the present study, there was no difference in the expression of c-Fos- and c-Jun-positive neurons at the same time-point, in the same spinal cord segment and same group. There was a high correlation with c-Fos and c-Jun expression.

In conclusion, following right C_7_ nerve root rhizotomy, c-Fos and c-Jun were expressed not only in ipsilateral C_7_ spinal gray matter, but also in ipsilateral C_5_ and C_6_ segments. Therefore, it was deduced that there is a neural pathway between ipsilateral adjacent cervical spinal cord segments and this may be one possible explanation as to why the radicular symptoms of cervical radiculopathy are not confined to the affected spinal cord segment, as shown by MRI.

## Abbreviations:

ABC: avidin-biotin-peroxidase complex;
MRI: magnetic resonance imaging;
IEG: immediate-early gene;
PBS: phosphate-buffered saline;
TBS: Tris-buffered saline
